# Long-Lasting Photocatalytic and Antimicrobial Activity of Cotton Dishcloths Finished with TiO_2_ Nanoparticles and Zinc Pyrithione

**DOI:** 10.3390/ma19132807

**Published:** 2026-07-02

**Authors:** Edyta Matyjas-Zgondek, Piotr Kulpiński, Elżbieta Skiba, Magdalena Popczyk, Andrzej S. Swinarew

**Affiliations:** 1 Department of Mechanical Engineering, Informatics and Chemistry of Polymer Materials, Lodz University of Technology, Żeromskiego 116, 90-924 Łódź, Poland; piotr.kulpinski@p.lodz.pl; 2 Institute of General and Ecological Chemistry, Lodz University of Technology, Żeromskiego 116, 90-924 Łódź, Poland; elzbieta.skiba@p.lodz.pl; 3 Faculty of Science and Technology, University of Silesia in Katowice, 75 Pułku Piechoty 1A, 41-500 Chorzów, Poland; magdalena.popczyk@us.edu.pl (M.P.); andrzej.swinarew@us.edu.pl (A.S.S.); 4 Institute of Sport Science, The Jerzy Kukuczka Academy of Physical Education, 40-065 Katowice, Poland

**Keywords:** photocatalytic, antimicrobial activity, self-cleaning cotton dishcloths, titanium dioxide, zinc pyrithione

## Abstract

This study assesses the durability of self-cleaning and antimicrobial activities of dishcloths made of 100% cotton woven fabric modified with TiO_2_ nanoparticles (NPs) and zinc pyrithione particles (ZnPt). The self-cleaning properties were measured as the ability to decompose staining particles via photocatalytic action under UV/VIS light irradiation and confirmed by colour measurements (K/S values, colour differences, and colour changes ΔL*, Δa*, Δb*). The SEM micrographs confirmed the long-term durability of the modification process, and ICP-OES analysis confirmed the presence of Ti and Zn elements on and within the fabric. The amounts of TiO_2_ and ZnPt decreased by 2.2–2.5 times after the first 5 washings and by 2.1–1.2 times after the subsequent 45 washings. Antimicrobial activity was tested against *Staphylococcus aureus* ATCC 6538, *Escherichia coli* ATCC 11229 and *Candida albicans* ATCC 10231. The results indicate that the material maintains excellent self-cleaning properties and good antimicrobial activity for up to 50 washings; however, activity against *E. coli* remains weak after 30 and 50 washings.

## 1. Introduction

Dishcloths, due to their high moisture absorption, relatively low cost, wide availability, and common use, are predominantly made of cotton. They are used to wipe dishes, hands, countertops, and other work surfaces. Cotton contains hydrophilic groups (such as hydroxyl groups) that can absorb moisture. Therefore, kitchen dishcloths, being particularly exposed to moisture and food residue, provide ideal conditions for microbial growth, especially for foodborne bacteria. Coliform, *Escherichia coli* (*E. coli*), *Staphylococcus aureus* (*S. aureus*), *Staphylococcus* spp., *Salmonella* spp., and *Listeria monocytogenes* are predominantly isolated from household dishcloths [1,2,3]. Thus, dishcloths are among the most microorganism-contaminated items in the kitchen and may contribute to the transfer of microorganisms to other surfaces and food, as well as to foodborne infections. Several decontamination methods for kitchen cloths are known, including hypochlorite and peroxide disinfection, high-temperature washing, soaking in vinegar or lemon juice, and microwaving, with microwaving being the most effective disinfection method [1]. Additionally, antimicrobial treatment can be used to prevent microbial growth on the dishcloth.

Moreover, kitchen dishcloths used in household conditions are often stained by various coloured liquids, such as coffee, tea, and fruit juices. Removing such stains poses many difficulties and often requires special cleaning methods, such as hypochlorite or peroxide discharge or high-temperature washing with a bath containing active oxygen stain removers. Unfortunately, some stains, especially on coloured kitchen cloths, may sometimes be impossible to remove. The problems associated with the formation and removal of coloured stains can be addressed by modifying textile surfaces with substances imparting self-cleaning properties. Ideally, the kitchen dishcloth would possess a surface characterised by both antimicrobial (to prevent bacterial growth) and self-cleaning properties (to remove coloured stains).

Zinc pyrithione (ZnPt) is a complex of zinc and pyrithione. ZnPt has antibacterial activity toward *E. coli*, *S. aureus*, *Klebsiella pneumoniae*, *Enterococcus faecium*, and *Enterococcus faecalis* [4] and antifungal activity toward *Pityrosporum ovale* and *Pityrosporum pachydermatis* [5]. ZnPt has been extensively used as an antifungal agent in shampoos, skin care products and in technical applications worldwide [6,7]. However, due to its classification as a substance of concern, ZnPt has been prohibited in cosmetic products in the European Union (EU) since 2022 [8,9]. The regulatory approaches differ globally. ZnPt may still be permitted outside the EU, such as in the United States [10,11]. In the European context, the current regulatory status of ZnPt in technical and industrial applications is complex and application-dependent, requiring consideration of specific legal frameworks.

Self-cleaning finishes can be split into three main categories: (1) superhydrophobic finishes, (2) superhydrophilic finishes and (3) photocatalytic finishes. Water droplets on superhydrophobic surfaces assume nearly perfect spherical shapes, allowing them to roll off and carry away all contaminants. Superhydrophobic finishes are achieved through the synergistic action of substances giving the fabric low surface tension and materials that create a complex, rough surface structure. On the contrary, the wettability of a superhydrophilic surface is extremely high. Water lies flat on the surface rather than forming droplets, cleaning the surface alongside the photocatalysis process [12,13]. Superhydrophobic finishes, due to their lack of surface wettability, cannot be used for finishing cotton fabrics intended for domestic textiles, such as cloths or kitchen towels. Self-cleaning finishes with superhydrophilic properties rely on the photocatalytic properties of semiconductors, which additionally exhibit photo-induced superhydrophilicity.

Photocatalysis is a process in which the rate of a chemical reaction changes under the action of ultraviolet, visible, or infrared radiation in the presence of a photocatalyst [14]. Semiconductors used in the photocatalytic processes are metal oxides (TiO_2_, Zn, SnO_2_, WO_3_, CeO_2_, ZrO_2_, Fe_2_O_3_), sulphides (Cd, ZnS, PbS, MoS_2_), selenides (CdSe) and tellurides (CdTe) [15]. TiO_2_ is the most widely used photocatalytic material in surface coatings such as glass, paint, cement, ceramics, textiles, etc. [16]. TiO_2_ occurs naturally in three crystalline forms: anatase, rutile, and brookite, where the anatase form shows the highest photocatalytic activity [17]. TiO_2_ NPs, under exposure to light (mainly UV light), are converted into positively charged holes (h^+^) and negatively charged electrons (e^−^). Electrons during contact with oxygen form a superoxide anion radical (O_2−_˙), whereas holes in the presence of water form hydroxyl radicals (˙OH) [18]. Generated reactive oxygen species (ROS) decompose organic contaminants, including coloured stains, through oxidation.

Recent studies [19,20,21,22,23] show an increasing interest in multifunctional textiles that combine self-cleaning properties, antimicrobial activity, and UV protection. However, most of these works focus on the initial performance of the materials, while industrial application and long-term durability are often omitted [23]. Many developed systems rely on laboratory techniques, such as sol–gel processing or in situ growth. These techniques are difficult to implement on an industrial scale [23]. Therefore, there is still a need to evaluate coatings produced using industrial finishing techniques under realistic conditions of use [23].

In previous studies, authors described the photocatalytic and antimicrobial activities of 100% cotton terry fabric finished with TiO_2_ N-doped NPs, ZnO NPs, and a binder system to improve durability. The material was characterised by significant antibacterial and antifungal activities, as well as greater photocatalytic efficiency, up to 15 washing cycles [21,22].

The aim of the present study is to evaluate the durability of the antimicrobial and self-cleaning properties of 100% cotton woven fabric and dishcloths made from it. The modification process was conducted under industrial conditions using a continuous padding-squeezing method in a bath containing dispersions of commercially available TiO_2_ nanoparticles (NPs) and zinc pyrithione particles (ZnPt). Particular attention was given to the retention of functional properties after multiple domestic washing cycles (up to 50 washes) and under selected conditions simulating domestic use (repeated washing cycles). To the best of our knowledge, few studies have addressed the long-term durability of TiO_2_/ZnPt systems applied using industrial techniques in real textile products, such as dishcloths.

## 2. Materials and Methods

### 2.1. Textile Modification

A 100% cotton woven fabric with a diagonal weave and a surface mass of 200 g/m^2^, made from chemically bleached and optically brightened cotton yarn, was purchased from ZWOLTEX Sp. z o.o., Zduńska Wola, Poland. TiO_2_ NPs (anatase crystal structure, average crystallite size of 10 nm and specific surface area > 250 m^2^/g) as a 20–22% water dispersion and zinc pyrithione particles (ZnPt) (high-fine particles) as a 48% water dispersion were purchased from Cinkarna (Celje, Slovenia) and The Thomson Research Associates Group (Canada), respectively. The crystalline structure of the TiO_2_ NPs in the commercial dispersion was verified by XRD analysis, conforming to the specification. No further structural analysis was performed, as both dispersions were used as received.

The cotton woven fabric was submitted to a functionalization process under industrial conditions using a continuous padding-squeezing method. The fabric was padded and squeezed in open-width form using the Bianco M15/2019 Padder Model RW2800/2600 FWMM (Bianco S.P.A., Alba, India) in a bath containing 10 g/L of nano TiO_2_ and 2.9 g/L of ZnP. The pressure of the squeezing shafts was 3 atm. Subsequently, the fabric was dried at 150 °C for 5 min in open-width form using the continuous tumbler dryer Penteck Energy X-Stream 3000/2800 (Penteck Textile Machinery Srl, Alba, Italy). All finishing processes were carried out in ZWOLTEX Sp. z o.o., Zduńska Wola, Poland. The finishing process is protected by Polish patent No. 235990. Next, a 50 × 70 cm dishcloth was made from modified cotton fabric.

#### Textile Material Characterisation

Changes in the surface topography of finished fabric before and after laundering were estimated using a Scanning Electron Microscope (SEM) VEGA3-SBU (Tescan, Brno, Czech Republic). The distribution of the elements on the fabric’s surface was estimated before and after 5 washings. The procedure for sample preparation and measurement was described in our previous works [21,22].

The durability of the obtained properties was tested after multiple washing and drying cycles. A household automatic washing machine (Indesit, Italy) with a 5 kg load capacity and typical commercial washing powder for white goods from a reputable company was used to carry out the washing process. A single washing and drying process was as follows:

- Washing powder concentration of 5 g/L;
- Washing temperature 60 °C, time 30 min, multiple rinses in cold water, spinning at 1000 rpm, ready-to-use washing machine program for cotton goods;
- Air-drying by hanging.

The dried fabric was subjected to the subsequent washing and drying process.

The designations of the tested cotton woven fabrics are shown in Table 1.

The ability of a cotton fabric surface functionalised with photocatalytic compounds to decompose staining particles was tested. To evaluate the self-cleaning properties, blackcurrant nectar containing 25% blackcurrant juice (Hortex Sp. z o.o., Warszawa, Poland) was selected. The self-cleaning properties were evaluated according to a procedure described previously [21,22].

The light reflectance R (%) was measured over a range of 400–700 nm. The self-cleaning action was evaluated by comparing the RK/S values, colour differences (E*), and colour changes (ΔL*, Δa*, Δb*) between irradiated and unirradiated stained samples. K/S values were determined according to the well-known Kubelka–Munk equation [24]. Finally, the K/S reduction (R_K/S_) values were calculated using the following formula:

R_(K/S)_ = [1 − (K/S)_aw_/(K/S)_bw_)] · 100%

where (K/S)_bw_ is the value measured for stained samples before UV/VIS light irradiation, and (K/S)_aw_ is the value measured for stained samples after UV/VIS light irradiation.

Quantitative determination of titanium (Ti) and zinc (Zn) concentrations in the modified goods before and after multiple washing/drying processes was carried out using the Inductively Coupled Plasma–Optical Emission spectrometer ICP-OES (PlasmaQuant PQ 9000, Analytik Jena, Jena, Germany). Before the measurements, 0.2 g of fabric samples were digested in a two-step procedure using an Anton Paar Multiwave 3000 microwave reaction system (Anton Paar GmbH, Graz, Austria). The first step was digestion with a mixture of concentrated acids HNO_3_ and HF applied together with H_2_O_2_ (30%) (3:1:1, *v*/*v*). The next step was complexation with a saturated H_3_BO_3_ solution, which complexes free fluoride ions and facilitates the dissolution of precipitated fluorides.

Based on the Ti and Zn concentrations, the content of the modifier per single modified dishcloth was calculated according to the following formulas:

TiO_2_ = 1.33 · C_Ti_ · m_dc_

ZnPt = 4.86 · C_Zn_ · m_dc_

% R_TiO2(ZnPt)_ = [1 − (TiO_2_(ZnPt)_aw_/TiO_2_(ZnPt)_bw_)] · 100

where

TiO_2_ and ZnPt are the calculated contents of modifier particles (TiO_2_ or ZnPt) in the fabric, respectively.

C_Ti_ and C_Zn_ are the quantitatively determined concentrations of Ti or Zn in the fabric, respectively.

m_dc_ is the mass of one dishcloth with dimensions of 50 × 70 cm.

% R_TiO2(ZnPt)_ is the percentage release of modifier particles (TiO_2_ or ZnPt) from the fabric.

bw and aw refer to measurements taken before washing and after washing, respectively.

Antibacterial and antifungal properties were tested at the Laboratorium Badań Włókienniczych Wyrobów Medycznych, Instytut Włókiennictwa, Poland. Antibacterial activity was assessed using the absorption method in accordance with PN-EN ISO 20743:2013-10 [25] while antifungal activity was assessed in accordance with AATCC Test Method 100-2004 [26]. *S. aureus* (ATCC 6538) and *E. coli* (ATCC 11229) were used for antibacterial tests, and *C. albicans* (ATCC 10231) for antifungal tests.

## 3. Results and Discussion

The objective of the study was to evaluate the durability of self-cleaning and biological activities in 100% cotton woven fabrics. Cotton fabric was surface functionalised under industrial conditions using a patented finishing technology and commercial dispersions of TiO_2_ and ZnPt particles. The concentrations of commercial modifiers were selected after preliminary studies.

**Figure 1:**
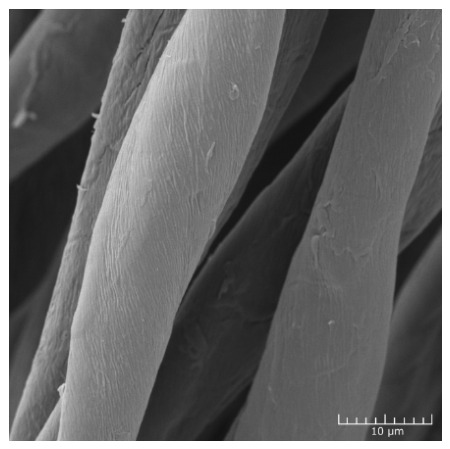
SEM image of untreated fabric (magnification ×10,000).

**Figure 2:**
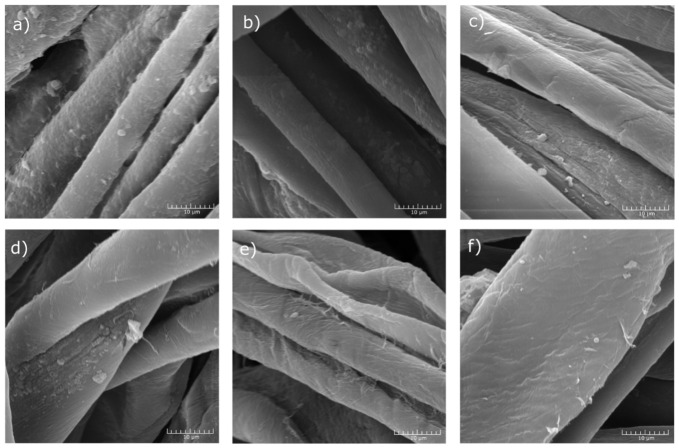
SEM images of modified fabric (magnification ×10,000): (**a**) before washing, (**b**) after 5 washing/drying cycles, (**c**) after 10 washing/drying cycles, (**d**) after 20 washing/drying cycles, (**e**) after 30 washing/drying cycles, and (**f**) after 50 washing/drying cycles.

Scanning electron microscopy (SEM) enables observation of the topography of modified cotton fabric before and after multiple washing and drying cycles. Figure 1 and Figure 2 show the SEM micrographs (with a magnification of ×10,000) of untreated and functionalised cotton fabric, respectively.

High-magnification SEM imaging of natural fibres, such as cotton, is highly susceptible to electron-beam-induced thermal degradation and structural loss. To preserve the integrity of the observed fibre morphology and prevent the formation of artificial artefacts caused by beam damage, we maintained a magnification level that ensured the most realistic representation of the surface.

Additional material can be observed on the surface of all modified fabrics (Figure 2a–f) compared to the unmodified fabric surface (Figure 1). It results from the deposition of TiO_2_ and ZnPt particles onto the surface of cotton fibres during the padding-squeezing finishing process. Dozens of particles, almost homogeneously distributed, are observed on the surface of the modified material before and after 5 washing and drying cycles. However, the irregular morphology of particle grains may be a consequence of the agglomeration of individual modifier particles. As expected, a decrease in the number of modifier particles on the fibre surface is observed after subsequent washing cycles (from 10 to 50 washings—Figure 2c–f), resulting from particles leaching out during laundering processes. Several modifier particles of different sizes and shapes can be seen on the surface of selected fibres after 10 and 20 washing cycles, whereas only single particles are present on the fibres’ surface after 30 and 50 washing and drying cycles. Also, partial damage to the cotton fibres’ surface can be seen (Figure 2e,f). Unfortunately, the SEM’s magnification did not allow the entire set of modifier particles present on the fibre surface to be shown.

**Figure 3:**
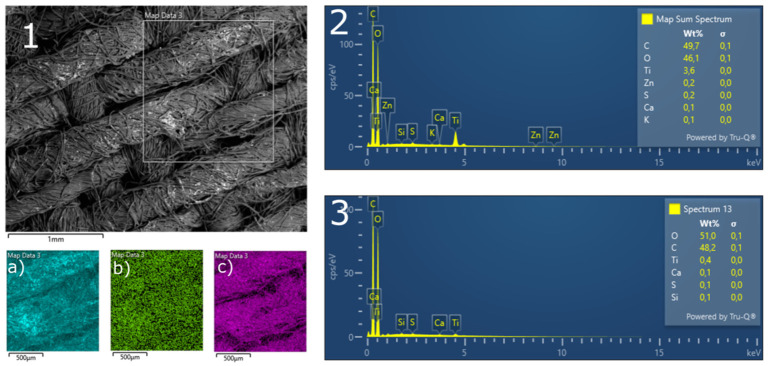
EDX-mapping of modified fabric before washing, (**1**)—modified fabric, (**a**)—titanium, (**b**) zinc, and (**c**) sulfur; and EDX spectra of (**2**)—modified fabric before washing and (**3**)—modified fabric after 5 washings.

To verify the distribution of modifiers on the fibre surface, EDX analysis was performed. Elemental mapping (Figure 3(1,a–c)) confirmed the presence and relatively uniform distribution of Ti, Zn, and S across the analysed area of unwashed samples. The Ti signal showed local areas of higher intensity, which are compatible with the presence of TiO_2_ agglomerates observed in SEM images.

Quantitative EDX analysis showed surface concentrations of approximately 3.6 wt% Ti, 0.2 wt% Zn, and 0.2 wt% S for the unwashed sample (Figure 3(2)). After repeated washing cycles (Figure 3(3)), the surface concentration of Ti decreased significantly (to ca. 0.4 wt%), while S remained at 0.1 wt%, and Zn was below the detection limit. This indicates significant removal of ZnPt from the fibre surface.

The self-cleaning properties of the finished fabric were evaluated. The colour changes between irradiated and unirradiated samples were assessed using R_K/S_, colour changes (ΔL*, Δa*, Δb*) and the colour differences (ΔE*). The ΔL* value refers to the difference in lightness from darker (−ΔL*) to lighter (+ΔL*), whereas Δa*and Δb* values refer to red/green differences from greener/less red (−Δa*) to redder/less green (+Δa*) and yellow/blue differences from bluer/less yellow (−Δb*) to yellower/less blue (+Δb*), respectively. The K/S value is linearly related to colourant concentration in the fabric, and ΔE* determines the total colour differences (the higher the ΔE* value, the greater the colour difference). The organoleptic observation of colour changes is not straightforward, as each observer’s perception of colour is unique. It can be assumed that a typical observer perceives the colour difference as follows: 0 ≤ ΔE* < 1 no notice of the colour difference; 1 ≤ ΔE* < 2 the colour difference noticed by a qualified observer, 2 ≤ ΔE* < 3.5 the colour difference is noticed by an unqualified observer, 3.5 ≤ ΔE* < 5 notice of a clear colour difference, ΔE* > 5 impression of two different colours.

The results of self-cleaning properties are shown in Table 2. It is indicated, based on R_K/S_ and ΔL* estimations, that all stained samples became lighter after exposure to UV/VIS light (ΔL* assumes positive values). It should be noted that all samples modified after light exposure are considerably lighter than the unmodified ones. An almost 70% K/S reduction (R_K/S_) was observed in the finished fabric after 5 washing cycles, compared to a ca. 46% reduction on the unwashed modified sample. Enhancement of photocatalytic properties may result from the removal of larger particles from the fabric surface. The R_K/S_ values for all modified samples are ca. 10 times greater (ranging from 9.2 for sample 2 to 13.9 for sample 3) than that of the control one, confirming the decomposition of colour compounds. Significant colour changes are observed in all samples, but the light-exposed, modified samples (before and after washing) appear to be two different colours (ΔE* > 5). Immense hue shifts from red to yellow (negative a* and positive b* values), resulting from the photocatalytic degradation of colour compounds, are shown for all finished samples. On the contrary, the untreated fabric shows only a slight colour difference after UV/VIS light exposure (ΔE* ca. 1.8), indicating a lack of photocatalytic properties. However, colour measurements indicated that the sample was slightly darker, less red, and yellower after UV/VIS irradiation.

**Figure 4:**
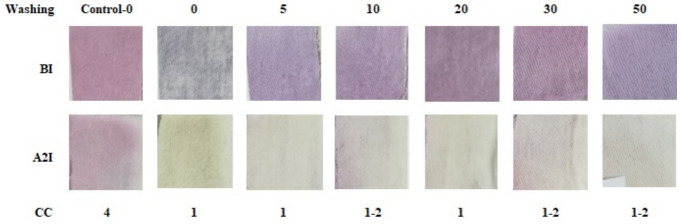
Pictures of stained samples before (**top**) and after (**bottom**) UV/VIS light exposure. BI—before irradiation, A2I—after 2 h and 15 min of irradiation, CC—the degree of change in colour.

Figure 4 shows images of stained samples (control and modified) before and after 2 h and 15 min of UV/VIS light exposure.

Prominent shade changes (significantly yellower and much less red) are visible in all light-irradiated modified samples, even in fabric subjected to 50 washing/drying cycles. However, the unwashed modified sample appears more lime-green, while samples subjected to 30 and 50 washes appear bluer. In contrast to ΔL* estimation, the irradiated stained control sample appears slightly lighter, with a slight shift toward less red, according to organoleptic assessment. The degree of change in colour (CC) for stained irradiated samples, according to greyscales, was calculated from colour measurements. It allows us to eliminate the inaccuracy of organoleptic assessment. The CC parameter was calculated as the degree of greyscale from 1 (great change in colour—excellent self-cleaning properties) to 5 (no colour change—lack of self-cleaning properties). The CC for all finished samples ranged from 1 to 1–2, indicating strong self-cleaning properties of the modified fabric. In contrast, the CC of the control sample, estimated at 4, indicates a lack of self-cleaning properties.

Table 3 shows the average concentration of Ti and Zn in cotton fabric determined by ICP-OES measurements, as well as the calculated amount of modifier in one dishcloth and the amount of modifier washed out from one dishcloth after washing cycles.

**Figure 5:**
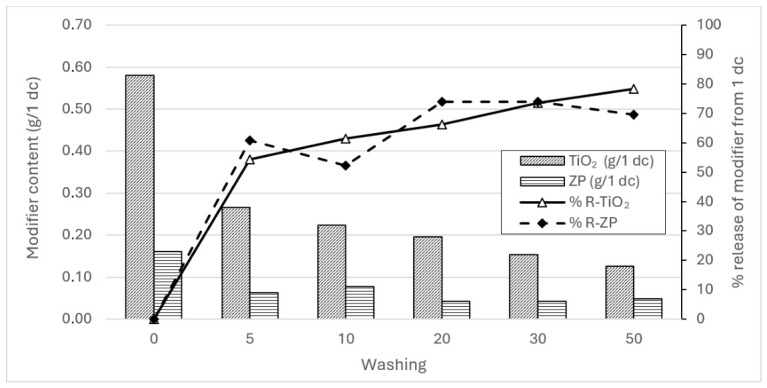
TiO_2_ NP and ZnPt concentrations on and in the cotton fibres, as stated by ICP measurements and percentage release of modifiers from textile goods.

The quantitative results of modifiers concentration in the cotton fabric, as expected, show a reduction in the content of both modifiers (TiO_2_ and ZnPt) after the subsequent washing and drying cycles. It can be stated that ca. 54% and ca. 60% of the modifier particles (TiO_2_ and ZnPt, respectively) deposited on and into cotton fibres were released from dishcloths during the first 5 washings. Based on our long-term experience with the evaluation of silver-finished textiles [27,28], we can assume that most of the modifier particles were released during the 1st washing cycle. We observed similar results during our previous work [22]. The level of ZnPt particles release (Figure 5) remains almost stable (almost no release of ZnPt) from the 20th to 50th washings, whereas a small amount of TiO_2_ is still leaking out from the cotton fabric during the 5th to 50th washings. The total loss of TiO_2_ NPs from the dishcloth across all 50 washing cycles is ca. 0.45 g, which represents ca. 78% of the total amount of TiO_2_ introduced into cotton fibres during the finishing process. In the case of ZnPt, total release amounted to ca. 0.11 g, which constitutes ca. 70% of the total ZnPt amount in the cotton fabric. Average loss of TiO_2_ NPs and ZnPt modifiers during subsequent 50 washing and drying cycles throughout a single washing amounts to ca 0.009 g/1 dc for TiO_2_ NPs and ca. 0.002 g/1 dc for ZnPt. It must be underscored that the average release of both modifiers during a single wash after the 5th wash amounts to ca. 0.003 g/1 dc. It should be emphasised that only a small amount of TiO_2_ NPs (ca 0.27 g/m^2^—amount after 50 washes) gives the fabric surface extremely strong self-cleaning properties. Although laundering is a major source of NPs emissions into the environment, the potential impact of sweat and mechanical friction (e.g., Martindale test) remains an important direction for future evaluation. The duration of skin contact with the kitchen dishcloth differs from that of direct-wear garments, thereby reducing the potential risk of exposure. The application of binders or cross-linking agents during the finishing process could improve the fixation of NPs with the textile structure. This would reduce initial NPs leaching and extend the lifetime of the items under repeated laundering cycles. Future research should explore such strategies.

The release of NPs requires a careful evaluation of their environmental and regulatory implications. NPs incorporated into commercial products are released into the environment, and their effects on living organisms, such as plants, should be thoroughly investigated [29,30]. Their impact depends on particle properties, plant species, and exposure conditions, leading to both beneficial and adverse outcomes [31,32,33]. While low concentrations of TiO_2_ can promote seed germination and enhance photosynthesis [34,35], the negative effects at high levels cannot be neglected and require further investigation [36].

In the case of ZnPt, it should be noted that it is a biologically active substance with a complex regulatory status in the EU [9]. While its use in cosmetics is prohibited [8], its application in textiles and industrial applications remains subject to strict regulation. The present study focuses on material durability rather than on regulatory compliance. As only the total zinc content was determined, no direct conclusions can be drawn regarding extractable zinc, which limits the assessment of dermal exposure and compliance with certification schemes based on extractable substances. However, the observed reduction in ZnPt content after initial washing cycles suggests the removal of loosely bound particles, which may be beneficial from a safety perspective.

Our results show the durability of the coating. However, further studies on extractability, washing-induced release, and long-term environmental impact are required to fully assess the safety of this technology.

The antimicrobial properties of functionalised dishcloths, before and after several washings, are presented in Table 4. The antimicrobial efficacy was assessed as a biostatic activity A and is interpreted according to ISO 20743 (Annex F) as follows: A < 0.5—lack of activity; 0.5 ≤ A < 2—slight activity; 2 ≤ A < 3—significant activity; and A ≥ 3—strong activity. A value of A = 1 corresponds to a one-log reduction in the microbial population [21].

The modified cotton dishcloths exhibit strong antimicrobial activity against *S. aureus* and *E. coli* strains for up to 20 washing cycles at 60 °C. While significant antibacterial activity was maintained against *S. aureus* after 30 to 50 wash cycles, the efficacy against *E. coli* weakened significantly (A = 0.69 and 1.27). It must be noted that all functionalised dishcloths (before and after washings) show strong activity against *C. albicans*. Furthermore, at a concentration of only ca. 50 mg ZnPt/1 dc, the system enables strong to excellent antimicrobial activity against *S. aureus* and *C. albicans*, but only slight activity against *E. coli*.

This study focuses on the assessment of hybrid finishing applied under industrial conditions, rather than on analysing the action of individual components (coatings consisting solely of TiO_2_ or ZnPt). This approach is important for evaluating functional durability in an industrial context.

The antimicrobial activity appears to result from two complementary pathways: the light-induced oxidative stress caused by TiO_2_ and the “dark-active” inhibitory effect of ZnPt. This combination enables sustained control of microorganisms even when photocatalytic activity is limited by light availability. Although the quantitative contribution of each pathway remains to be determined, the results indicate the presence of a dual-action mechanism effective in textile applications.

Furthermore, the assessment of the antimicrobial activity of photocatalytic systems involves methodological challenges that may limit the direct evaluation of photocatalytically induced antimicrobial effects [37]. In the present study, the standard test methods (ISO 20743, AATCC 100) do not allow for direct assessment of the antimicrobial activity induced by photocatalysis. Therefore, while ROS-related processes may contribute to the overall efficiency of the system, their contribution cannot be directly distinguished under the experimental conditions used.

## 4. Conclusions

The results of the study indicate that the deposition of TiO_2_ NPs and ZnPt particles using the padding-squeezing technique is a simple and effective approach to achieving photocatalytic and antimicrobial properties in 100% cotton woven fabric and the dishcloths made of it.

SEM micrographs confirmed that both modifiers (TiO_2_ and ZnPt) remain on the fabric surface after multiple washing and drying cycles (even after 50 washes). ICP analysis also validated the presence of modifier particles on and in the cotton fibres. The amounts of TiO_2_ and ZnPt decreased by 4.6 and 3.3 times, respectively, compared to the initial level.

The results indicate that TiO_2_/ZnPt finishing provides durable self-cleaning properties and partial antimicrobial activity after repeated washing cycles. However, antimicrobial effectiveness, particularly against E. coli, decreases with increasing numbers of washing cycles.

From an application perspective, further optimisation is required to improve particle retention and balance functional performance with safety and environmental considerations. In particular, additional studies on extractable Zn and washing-induced release are necessary to assess the suitability of ZnPt-treated textiles for practical use.

## Figures and Tables

**Table 1:** Designations of the tested dishcloths.

Washing	Sample Description
Control-0	control dishcloth made of 100% cotton woven fabric (without modification)
0	dishcloth made of 100% modified cotton woven fabric (before washing)
5	dishcloth made of 100% modified cotton woven fabric (5 washings)
10	dishcloth made of 100% modified cotton woven fabric (10 washings)
20	dishcloth made of 100% modified cotton woven fabric (20 washings)
30	dishcloth made of 100% modified cotton woven fabric (30 washings)
50	dishcloth made of 100% modified cotton woven fabric (50 washings)

**Table 2:** Self-cleaning properties.

Washing	R_K/S_ (λ = 540 nm) (%)	ΔE*	ΔL*	Δa*	Δb*
Control-0	5.0	1.781	−0.707	−0.138	1.629
0	46.15	14.514	4.626	−1.363	13.690
5	69.44	14.760	9.540	−3.727	10.629
10	53.85	10.507	6.953	−2.335	7.524
20	64.29	13.579	8.973	−4.147	9.311
30	55.26	11.907	7.067	−2.011	9.063
50	55.0	10.487	7.193	−1.434	7.495

**Table 3:** Average concentration of Ti and Zn in the cotton fabric as stated by ICP-OES measurement (mean ± SD; n = 3), as well as the calculated amount of modifier in one dishcloth and the amount of modifier washed out from one dishcloth after repeated washing cycles.

Washing	Titanium			Zinc		
	Ti (g/kg)	TiO_2_ (g/1 dc)	R	Zn (g/kg)	ZnPt (g/1 dc)	R
Control-0	0.016	-	-	0.012	-	-
0	6.20 ± 0.05	0.581	-	0.46 ± 0.01	0.161	-
5	2.84 ± 0.12	0.266	0.315	0.19 ± 0.01	0.063	0.098
10	2.41 ± 0.01	0.224	0.042	0.23 ± 0.03	0.077	∼0
20	2.12 ± 0.03	0.196	0.028	0.12 ± 0.01	0.042	0.035
30	1.68 ± 0.01	0.154	0.042	0.11 ± 0.01	0.042	0
50	1.36 ± 0.01	0.126	0.028	0.13 ± 0.01	0.049	∼0

R is the amount of modifier released from one dishcloth, calculated after each selected washing cycle relative to the previous measurements. 1 dc means one dishcloth.

**Table 4:** Antimicrobial properties against *S. aureus* and *E. coli* bacteria strains and *C. albicans* fungi.

Washing	Bacteria Strains		*C. albicans*
	*S. aureus*	*E. coli*	
0	5.3	4.6	3.6
5	4.0	5.3	3.8
10	5.2	5.3	3.5
20	5.3	5.0	3.6
30	2.76	0.69	3.9
50	2.97	1.27	3.5

## Data Availability

The original contributions presented in this study are included in the article. Further inquiries can be directed to the corresponding author.

## References

[1] Gillies E. (2021). Determining the most effective common household disinfection method to reduce the microbial load on domestic dishcloths: A pilot study. Environ. Health Rev..

[2] Gerba C.P., Tamimi A.H., Maxwell S., Sifuentes L.Y., Hoffman D.R., Koenig D.W. (2014). Bacterial Occurrence in Kitchen Hand Towels. Food Prot. Trends.

[3] (2011). IFH—Home Hygiene & Health The Infection Risks Associated with Clothing and Household Linens in Home and Everyday Life Settings, and the Role of Laundry. http://www.ifh-homehygiene.org/IntegratedCRD.nsf/eb85eb9d8ecd365280257545005e8966/d0e3b0f361079f1780257865003d43b1?OpenDocument.

[4] Blanchard C., Brooks L., Ebsworth-Mojica K., Didione L., Wucher B., Dewhurst S., Krysan D., Dunman P.M., Wozniak R.A.F. (2016). Zinc Pyrithione Improves the Antibacterial Activity of Silver Sulfadiazine Ointment. Ther. Prev..

[5] Cutsem J.V., Gerven F.V., Fransen J., Schrooten P., Janssen P.A. (1990). The in vitro antifungal activity of ketoconazole, zinc pyrithione, and selenium sulfide against Pityrosporum and their efficacy as a shampoo in the treatment of experimental pityrosporosis in guinea pigs. J. Am. Acad. Dermatol..

[6] Kumari N., Bhattacharya S.N., Das S., Datt S., Singh T., Jassal M., Agrawal A.K. (2021). In Situ Functionalization of Cellulose with Zinc Pyrithione for Antimicrobial Applications. ACS Appl. Mater. Interfaces.

[7] Rayana H.B., Dhouib S., Babay A., Chaouch W., Djelassi B., Zouari R. (2022). Study of the Antibacterial Efficiency of Zinc Pyrithione Treated Cotton Fabric for Shoe Insoles: Optimizing the Zinc Content and Developing a Spectrophotometric Method. J. Nat. Fibers.

[8] Regulation (EU) 2021/1902 of the European Parliament and of the Council of 29 October 2021 amending Annexes II, III and V to Regulation (EC) No 1223/2009 of the European Parliament and of the Council as Regards the Use in Cosmetic Products of Certain Substances Classified as Carcinogenic, Mutagenic or Toxic for Reproduction (Text with EEA Relevance). https://eur-lex.europa.eu/eli/reg/2021/1902/oj/eng.

[9] Opinion on Zinc Pyrithione (ZPT) (P81) CAS N° 13463-41-7—Submission III (SCCS/1614/19 Final Opinion). https://health.ec.europa.eu/document/download/aa535110-c020-4924-8507-5f867adc9972_en.

[10] U.S. Food and Drug Administration Over-the-Counter (OTC) Monograph M032: Drug Products for the Control of Dandruff, Seborrheic Dermatitis, and Psoriasis for Over-the-Counter Human Use (Posted 16 December 2021). https://www.accessdata.fda.gov/drugsatfda_docs/omuf/monographs/OTC%20Monograph_M032-Drug%20Products%20for%20the%20Control%20of%20Dandruff%20Seborrheic%20Dermatitis%20and%20Psoriasis%2012.16.2021.pdf.

[11] U.S. Environmental Protection Agency (EPA) Pesticide Product Label System (PPLS), Registration No. 92760-11. https://ordspub.epa.gov/ords/pesticides/f?p=PPLS:8:16897659424880::NO::P8_PUID,P8_RINUM:522362,92760-11.

[12] Krishna M.G., Vinjanampati M., Purkayastha D.D. (2013). Metal Oxide Tin films and nanostructures for self-cleaning applications: Current status and future prospects. Eur. Phys. J. Appl. Phys..

[13] Saad S.R., Mahmed M., Abdullah M.M.M.A.B., Sandu A.V. (2016). Self-Cleaning Technology in Fabric: A Review. IOP Conf. Ser. Mater. Sci. Eng..

[14] Wu J., Zheng W., Chen Y. (2022). Definition of photocatalysis: Current understanding and perspective. Curr. Opin. Green Sustain. Chem..

[15] Pelizzetti E., Carlin V., Minero C., Grätzel M. (1991). Enhancement of the rate of photocatalytic degradation on TiO_2_ of 2-chlorophenol, 2,7-dichlorodibenzodioxin and atrazine by inorganic oxidizing species. New J. Chem..

[16] Foster H.A., Ditta I.B., Varghese S., Steele A. (2011). Photocatalytic disinfection using titanium dioxide: Spectrum and mechanism of antimicrobial activity. Appl. Microbiol. Biotechnol..

[17] Luttrell T., Halpegamage S., Tao J., Kramer A., Sutter E., Batzill M. (2014). Why is anatase a better photocatalyst than rutile?—Model studies on epitaxial TiO_2_ films. Sci. Rep..

[18] Nuaim M.A.A., Alwasiti A.A., Shnain Z.Y. (2023). The photocatalytic process in the treatment of polluted water. Chem. Pap..

[19] Salama K.F., AiJindan R., Alfadehel A., Akhar S., Al-Suhaimi E.A. (2024). Enhanced antimicrobial performance of textiles coated with TiO_2_ nanoparticles. J. Ind. Text..

[20] Pakdel E., Daoud W.A., Kashi S., Gashti M.P., Wang X. (2025). Superhydrophilic self cleaning fabric with enhanced antibacterial and UV protection properties. Cellulose.

[21] Gutarowska B., Szulc J., Matyjas-Zgondek E., Kulpiński P., Pielech-Przybylska K., Rygala A., Jachowicz A., Rutkowski E. (2020). Cotton terry textiles with photo- and bio-activity in a model study and real conditions. Materials.

[22] Gutarowska B., Matyjas-Zgondek E., Kulpiński P., Mroczyńska-Florczak M., Rutkowski E. (2021). Long-Lasting Photocatalytic and Antimicrobial Activity of Cotton Towels Modified with TiO_2_ and ZnO Nanoparticles. Catalysts.

[23] Lee J.-C., Huh M.-W., Hou Y.-L., Kim W.-J. (2025). Complex Challenges in the Textile Industry and Potential Solutions in Photocatalytic Coating Technology: A Systematic Literature Review. Materials.

[24] Pruś S., Kulpiński P., Matyjas-Zgondek E. (2021). Comparison of the effects of the cationization of raw, bio- and alkali-scoured cotton knitted fabric with different surface charge density. AUTEX Res. J..

[25] (2013). Textiles-Determination of Antibacterial Activity of Textile Products.

[26] (2004). Antibacterial Finishes on Textile Materials: Assessment of.

[27] Bacciarelli-Ulacha A., Rybicki E., Matyjas-Zgondek E., Pawlaczyk A., Szynkowska M.I. (2014). A New Method of Finishing of Cotton Fabric by in Situ Synthesis of Silver Nanoparticles. Ind. Eng. Chem. Res..

[28] Bacciarelli-Ulacha A., Matyjas-Zgondek E., Puchowicz D., Cieślak M. (2022). Comparison of Innovative Silver Nanoparticles Finishing Technologies to Obtain Antibacterial Properties of Cotton Fabric. Fibers Polym..

[29] Tong T., Wilke C.M., Wu J., Binh C.T.T., Kelly J.J., Gaillard J.F., Gray K.A. (2015). Combined Toxicity of Nano-ZnO and Nano-TiO_2_: From Single- to Multi nanomaterial Systems. Environ. Sci. Technol..

[30] Nthunya L.N., Mosai A.K., López-Maldonado E.A., Bopape M., Dhibar S., Nuapia Y., Ajiboye T.O., Buledi J.A., Solangi A.R., Sherazi S.T.H. (2025). Unseen threats in aquatic and terrestrial ecosystems: Nanoparticle persistence, transport and toxicity in natural environments. Chemosphere.

[31] Skiba E., Adamczyk-Szabela D., Wolf W.M., Singh V.P., Singh S., Prasad S.M., Chauhan D.K., Tripathi D.K. (2020). Metal based nanoparticles interactions with plants. Plant Responses to Nanomaterials: Recent Interventions and Physiological and Biochemical Responses.

[32] Kandhil U., Singh G., Rani A., Dang A.S., Giri S.K., Dhiman S.S., Verma N., Kumar A. (2025). Soil–microbe–plant continuum under ZnO and TiO_2_ nanoparticle stress: An insight into toxicological implications, risk evaluation and management strategies. Plant Nano Biol..

[33] Shah M.A., Shahnaz T., Zehab-ud-Din, Masoodi J.H., Nazir S., Qurashi A., Ahmed G.H. (2024). Application of nanotechnology in the agricultural and food processing industries: A review. Sustain. Mater. Technol..

[34] Li C.-C., Jhou S.-M., Li Y.-C., Ciou J.-W., Lin Y.-Y., Hung S.-C., Chang J.-H., Chang J.-C., Sun D.-S., Chou M.-L. (2022). Exposure to low levels of photocatalytic TiO_2_ nanoparticles enhances seed germination and seedling growth of amaranth and cruciferous vegetables. Sci. Rep..

[35] Skiba E., Pietrzak M., Michlewska S., Gruszka J., Malejko J., Godlewska-Żyłkiewicz B., Wolf W.M. (2024). Photosynthesis Governed by Nanoparticulate Titanium Dioxide. The *Pisum sativum* L. Case Study. Environ. Pollut..

[36] Tan W., Peralta-Videa J.R., Gardea-Torresdey J.L. (2018). Interaction of titanium dioxide nanoparticles with soil components and plants: Current knowledge and future research needs—A critical review. Environ. Sci. Nano.

[37] Cunliffe A.J., Askew P., Iredale G., Marchant A., Redfern J. (2024). Methods to assess antibacterial, antifungal and antiviral surfaces in relation to touch and droplet transfer: A review, gap-analysis and suggested approaches. Access Microbiol..

